# A Novel *CLTC::RPS6KB1* Fusion in a Poorly Differentiated Carcinoma Involving the Lung and Mediastinum

**DOI:** 10.1177/10668969251323935

**Published:** 2025-03-16

**Authors:** Mitchell Zhao, Nicholas Protopsaltis, Mina Sabet, Shulei Sun, Grace Lin, Farnaz Hasteh, Wei Song

**Affiliations:** 1 Department of Pathology and Laboratory Medicine, 8784 University of California San Diego, La Jolla, California, USA

**Keywords:** *RPS6KB1* fusion, poorly differentiated carcinoma involving lung

## Abstract

Cancer genetics studies have dramatically advanced the understanding of the molecular drivers in various types of neoplasms. This progress is also leading to the discovery of more new molecular agents to block those drivers, which has significantly improved cancer patient survival, especially in non-small cell lung cancer (NSCLC). However, in about 25% of NSCLC tumors molecular drivers are not yet known. Herein, we present a poorly differentiated carcinoma involving lung and mediastinum. Next generation sequencing-based RNAseq identified a novel fusion, *CLTC::RPS6KB1*, while no other known drivers were present.

## Introduction

Next generation sequencing (NGS) has played an integral role in oncology, allowing us to simultaneously sequence many genes to better characterize tumors and identify their critical mutations. Such mutations may be associated with drug resistance or make the patient eligible for specific, targeted treatments, opening the opportunity for more personalized treatment of cancer.^1^

Ribosomal protein S6 kinase β-1 (S6K1) is coded for by the *RPS6KB1* gene and plays an important role in cellular growth and metabolism via the mTOR pathway. S6K1 has been studied as a marker of mTOR pathway amplification, and the protein itself has been studied as a potential target for treatment.^2,3^

Here, we report a patient with a high-grade malignant neoplasm, most compatible with carcinoma, involving the lung and mediastinum in which we identify a novel *CLTC::RPS6KB1* fusion as the likely driver mutation. We then review the literature involving *RPS6KB1* and its role in oncogenesis.

## Patient Presentation

**Figure 1.**
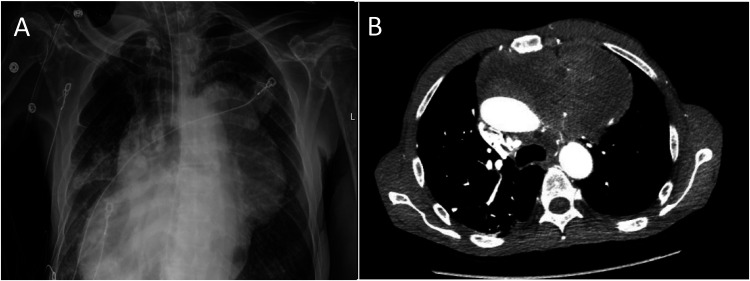
Chest X-ray demonstrating a large anterior superior mediastinal mass with displacement of the cardiac silhouette (A). CT scan showing the mass with extension to the left atrium, interatrial septum, lateral wall of the left ventricle, and left upper lobe of the lung (B).

A 63-year-old man presented to the emergency department with a chief complaint of progressive shortness of breath associated with fatigue and unintentional weight loss (over 40 lbs in the past 1.5 months). Physical examination was notable for cachexia and tachypnea, and the patient was admitted for further evaluation. A chest X-ray was performed, revealing a large mass centered in the anterior superior mediastinum displacing the heart to the right (Figure 1A). Further characterization of the mass by chest CT demonstrated the mass to measure at least 16.5 × 15 cm with heterogeneous enhancement, extending into the left upper lobe, pericardium, heart, and esophagus with encasement of the main pulmonary artery and aorta (Figure 1B). The remaining lung fields showed inflammatory/infectious changes. CT of the abdomen and pelvis showed no significant findings.

**Figure 2.**
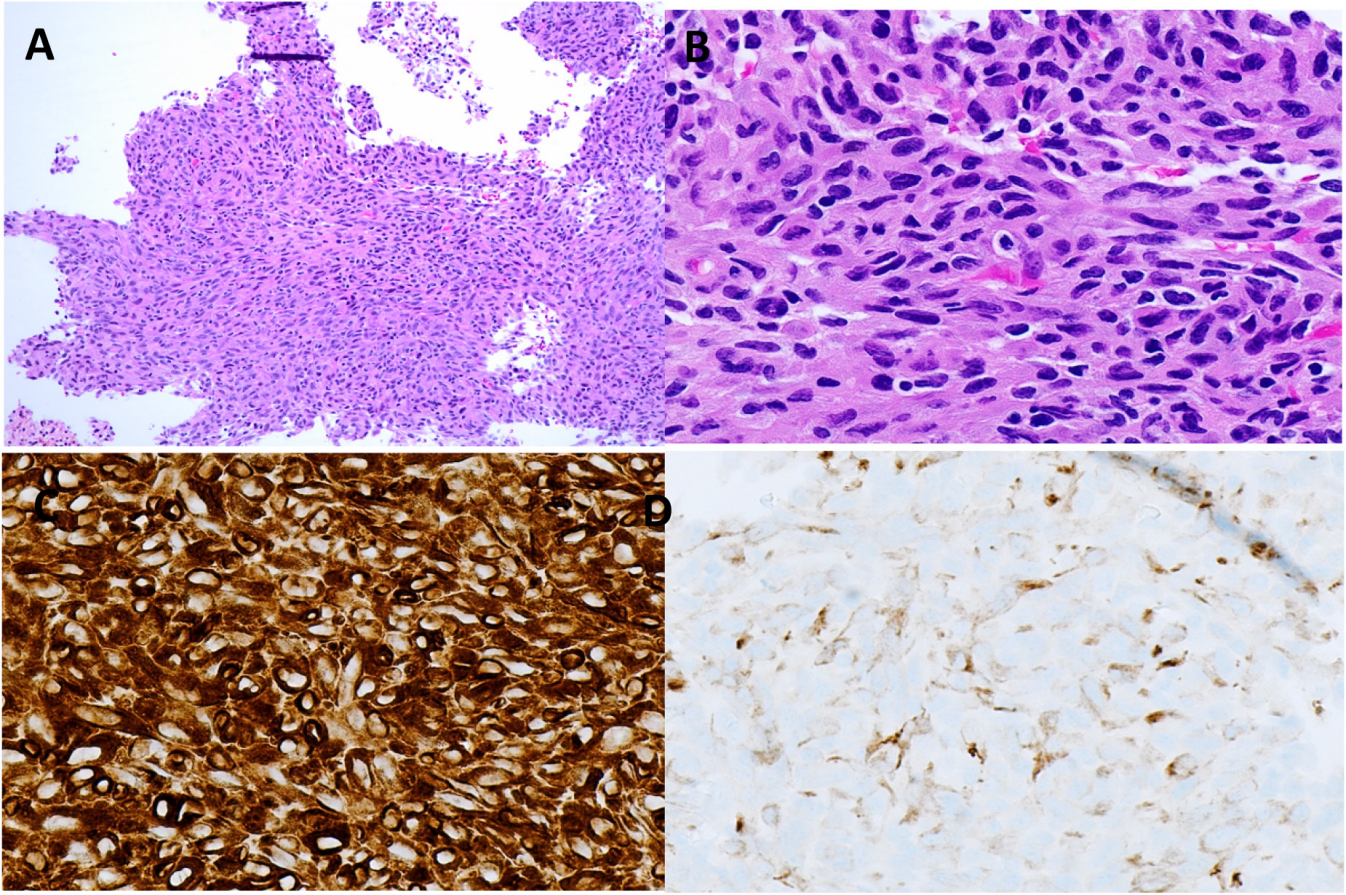
Microscopic examination of the mediastinal mass revealed a high-grade malignant spindle cells with abundant cytoplasm and a high mitotic rate. Extensive necrosis was identified (A, B). Immunohistochemistry demonstrated strong, diffuse staining for vimentin (C) and weak, patchy staining for pankeratin (D).

The patient underwent a percutaneous biopsy to sample the mediastinal mass, and samples were sent to surgical pathology and cytopathology. Microscopic evaluation of both specimens revealed a high-grade spindle cell neoplasm with marked nuclear pleomorphism and abundant mitoses in a background of extensive necrosis and lymphohistiocytic inflammation (Figure 2). Squamous and glandular differentiation were not identified. Immunohistochemical stains demonstrated tumor cells to be diffuse, strongly positive for vimentin and weak, patchy positive for pankeratin and KRT7. Tumor cells were negative for LCA, P63, P40, S100, SOX10, KIT, SALL4, MOC31, Claudin-4, INSM1, synaptophysin, desmin, ALK, EBER-ISH, HHV-8, and H3K27me3 (retained nuclear staining). Proliferation index by Ki-67 was 80%. PD-L1 testing by immunohistochemistry (22C3 clone) revealed a tumor proportion score of 70–80%. Despite an extensive workup, no definitive line of differentiation could be determined.

**Figure 3.**
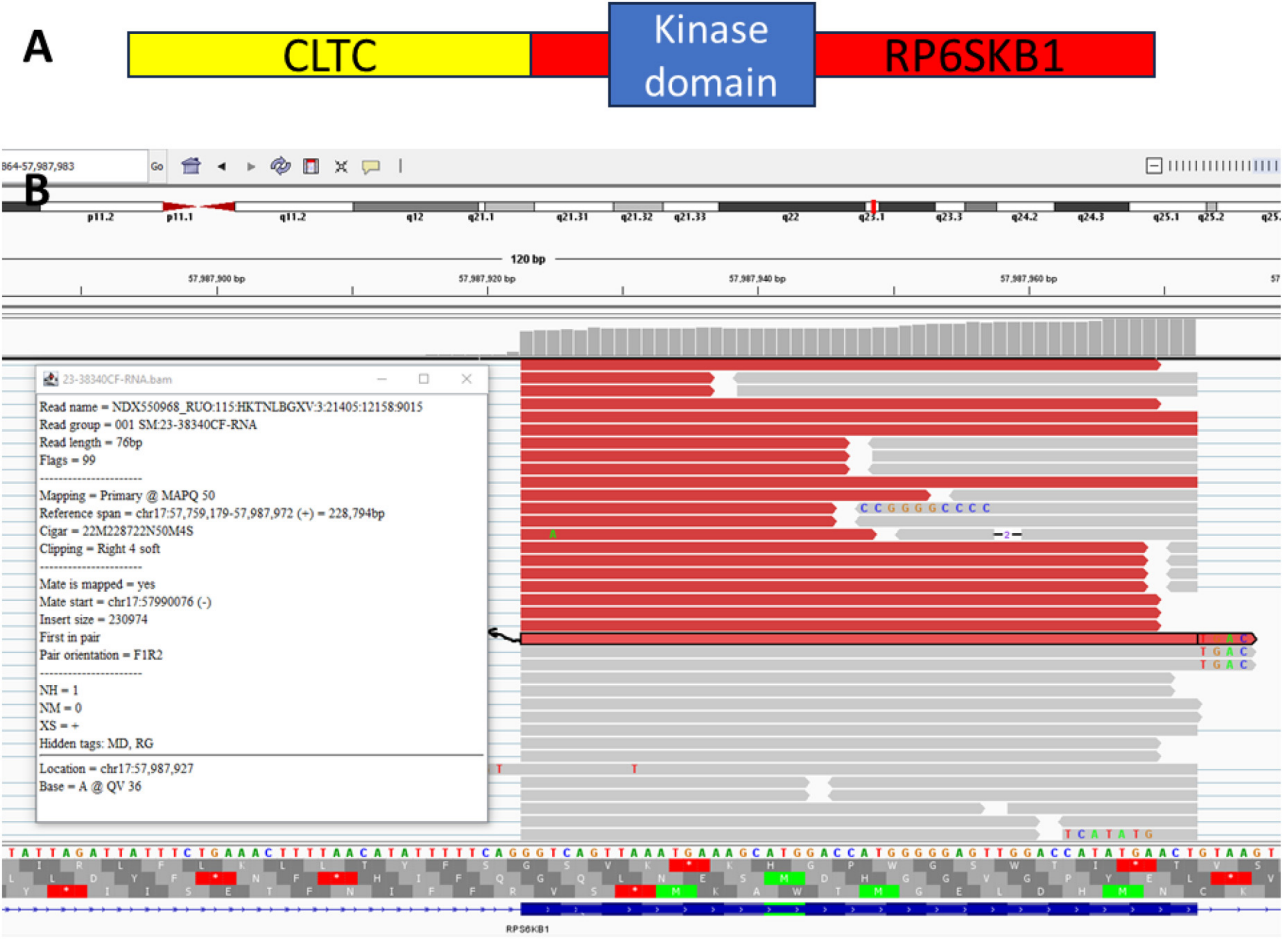
*CLTC::RP6SKB1* fusion protein. (A) It highlights the general structure of the fusion transcript with preservation of the kinase domain of *RP6SKB1*. (B) It outlines the reads obtained on NGS at the *RPS6KB1* side breakpoint.

Tissue from the biopsy was run on the TruSight Oncology 500 Assay (TSO-500, Illumina, San Diego, CA, USA). The panel identified a *CLTC::RPS6KB1* fusion rearranged at exon 2 of *RPS6KB1* that included the kinase domain of *RPS6KB1* (Figure 3). This fusion was predicted to be clinically significant. The tumor mutation burden was 14.9Muts/Mb. Additional molecular findings are summarized in Table 1. Overall, findings were determined to be most compatible with a poorly differentiated carcinoma.

**Table 1.** Summary of Molecular Findings (TSO-500).

Gene	Coding variant	Protein change	Annotation
*RPS6KB1*	-	*CLTC::RPS6KB1* fusion	clinically significant
*CDKN2A*	c.227-228insG	p.T77Hfs*43	clinically significant
*TP53*	c.818del	p.R273Lfs*72	clinically significant
*PAX7*	c.1423G > T	p.A475S	VUS
*VTCN1*	c.191T > A	p.L64H	VUS
*LRP1B*	c.9625 + 2T > G	-	likely clinically significant
*PDGFRA*	c.3008G > T	p.W1003L	VUS
*SNCAIP*	c.2225A > C	p.K742T	VUS
*INHBA*	c.764T > C	p.V255A	VUS

During this time, the patient was eventually upgraded to the intensive care unit due to progressive hypoxic respiratory failure requiring intubation. He was started on vancomycin and piperacillin/tazobactam for pneumonia and was subsequently switched to meropenem after sputum cultures grew out *Klebsiella pneumoniae*. Oncology was consulted, and the patient was started briefly on carboplatin/paclitaxel (treatment was started prior to PD-L1 results being available). Despite treatment, the patient continued to clinically decline, requiring both increased ventilator support as well as significant pressor support due to the development of shock. After discussions with the patient and his family, the decision was made to transition to comfort care.

## Discussion

*RPS6KB1* is located on chromosome 17 and codes for S6K1, a serine/threonine kinase immediately downstream of mTORC1 in the mTOR pathway. S6K1 has multiple isoforms due to the presence of multiple translational start sites and alternative splicing with p70-S6K1 being the most well-studied isoform. Important to this protein is the kinase domain which is involved in the protein's enzymatic activity as well as an N-terminal domain and C-terminal domain which are responsible for keeping the protein in an inactive state.^2^ S6K1 is activated by phosphorylation by mTOR and subsequently plays a role in multiple downstream pathways involving protein synthesis (via phosphorylation of ribosomal protein S6), transcription, translation, apoptosis inhibition, and cellular proliferation.^2–4^

There have also been studies looking at the role of S6K1 in lung cancer regarding treatment. In vitro studies have demonstrated that the inhibition of S6K1 signaling leads to increased radiosensitivity in lung cancer cell lines.^5,6^ S6K1 amplification has also been seen to be associated with resistance to EGFR tyrosine kinase inhibitors treatment resistance and lower recurrence-free survival in EGFR-mutant lung adenocarcinoma.^7^ This relationship between S6K1 and EGFR treatment resistance was similarly seen in a mouse model study demonstrating that S6K1 signaling allowed tumor cells to overcome EGFR inhibition through MDM2 upregulation. When S6K1 was inhibited, though, TKI treatment showed improved efficacy.^8^

While there are ample studies looking at *RPS6KB1*/S6K1 regarding amplification and activation in oncogenesis,^4,5,9–11^ there are conversely very few reports in the literature discussing *RPS6KB1* gene rearrangements in relation to malignancy. A recurrent *VMP1::RPS6KB1* fusion t(17;17)(q23.1;q23) has been reported in a series of lung adenocarcinoma tumors as well as in esophageal.^12,13^ For the lung cancer series, this particular fusion does preserve the kinase domain, suggesting a possible role in oncogenesis.^12^ *RPS6KB1* was not a target of fusion detection panel in some major commercial NGS pipeline, including Foundation One. This might explain why *RPS6KB1* fusion has not been reported in any non-small cell lung cancer tumor in any peer-reviewed literature.

Currently, most cancer treatment algorithms are structured around the cancer type. As a result, determining the optimal treatment regimens is difficult when the specific tumor type cannot be identified. In this instance, the tumor showed no definitive morphologic or immunohistochemical evidence of differentiation. Weak, patchy cytokeratin staining was suggestive of carcinoma, and the tumor ultimately had to be managed as a carcinoma of unknown primary and treated with general systemic chemotherapy. Due to the short timeframe between initiation of chemotherapy and the patient's death, efficacy of treatment and associated adverse events could not be adequately evaluated.

In these scenarios, the identification of a targetable molecular aberration can increase the repertoire of potential treatment options beyond the tumor type-specific algorithms, even if said treatment is not necessarily FDA-approved for a particular tumor type. Such treatments are usually both highly effective and result in fewer cytotoxicities than systemic chemotherapy due to their specificity in targeting specific pathways.^14^

Of note, this patient's management is complicated by the fact that the tumor's differentiation and site of origin cannot be resolved by standard pathologic evaluation. Overall, the tumor shows no specific morphologic features to suggest differentiation or site of origin, and the immunohistochemical profile is nonspecific. Given the tumor's involvement of both the mediastinum and lung, the differential diagnosis is broad, including but not limited to pulmonary carcinoma, thymic tumors, germ cell tumors, metastatic disease, and sarcomas. When taking the patient's age, significant smoking history, and imaging findings into account, the presentation is most concerning for pulmonary carcinoma, with morphology suggestive of a sarcomatoid carcinoma specifically. However, the biopsy material demonstrates no definitive evidence for epithelial differentiation by morphology or immunohistochemistry. While the tumor does show patchy keratin staining, the degree of staining on the biopsy is insufficient to be considered as reliable evidence of epithelial differentiation. A definitive diagnosis of pulmonary sarcomatoid carcinoma cannot be rendered here, especially since the tumor was never resected for comprehensive pathologic evaluation. Nevertheless, interdisciplinary evaluation is highly suggestive of a pulmonary carcinoma.

We report a previously undescribed *CLTC::RPS6KB1* fusion in a high grade, undifferentiated carcinoma of suspected pulmonary origin. This fusion likely serves as the driver mutation for the neoplasm through activation of the mTOR pathway given the lack of other identifiable driver mutations on molecular testing. These findings suggest that *RPS6KB1* should be evaluated not only as a marker for mTOR pathway activation but also as a target itself for novel targeted cancer treatments.^15^
